# Multifarious Trajectories in Plant-Based Ethnoveterinary Knowledge in Northern and Southern Eastern Europe

**DOI:** 10.3389/fvets.2021.710019

**Published:** 2021-10-14

**Authors:** Giulia Mattalia, Olga Belichenko, Raivo Kalle, Valeria Kolosova, Natalia Kuznetsova, Julia Prakofjewa, Nataliya Stryamets, Andrea Pieroni, Gabriele Volpato, Renata Sõukand

**Affiliations:** ^1^Department of Environmental Sciences, Informatics and Statistics, Ca' Foscari University of Venice, Venice, Italy; ^2^Institute of Environmental Science and Technology, Autonomous University of Barcelona, Barcelona, Spain; ^3^University of Gastronomic Sciences of Pollenzo, Bra, Italy; ^4^Institute for Linguistic Studies, Russian Academy of Sciences, St. Petersburg, Russia; ^5^Department of Medical Analysis, Tishk International University, Erbil, Iraq

**Keywords:** alternative and complementary veterinary medicine, animal husbandry, livestock, local ecological knowledge, plant-based remedies

## Abstract

Over the last century in the European context, animal production has been transformed by the dynamics of centralization and decentralization due to political and economic factors. These processes have influenced knowledge related to healing and ensuring the welfare of domestic animals. Therefore, our study aimed to document and compare current and past ethnoveterinary practices, and to identify trajectories in ethnoveterinary knowledge in study regions from both northern and southern Eastern Europe. In the summers of 2018 and 2019, we conducted 476 interviews, recording the use of 94 plant taxa, 67 of which were wild and 24 were cultivated. We documented 452 use reports, 24 of which were related to the improvement of the quality or quantity of meat and milk, while the other 428 involved ethnoveterinary practices for treating 10 domestic animal taxa. Cattle were the most mentioned target of ethnoveterinary treatments across all the study areas, representing about 70% of all use reports. Only four plant species were reported in five or more countries (*Artemisia absinthium, Hypericum* spp., *Linum usitatissimum, Quercus robur*). The four study regions located in Northern and Southern Eastern Europe did not present similar ethnoveterinary knowledge trajectories. Bukovinian mountain areas appeared to hold a living reservoir of ethnoveterinary knowledge, unlike the other regions. Setomaa (especially Estonian Setomaa) and Dzukija showed an erosion of ethnoveterinary knowledge with many uses reported in the past but no longer in use. The current richness of ethnoveterinary knowledge reported in Bukovina could have been developed and maintained through its peculiar geographical location in the Carpathian Mountains and fostered by the intrinsic relationship between the mountains and local pastoralists and by its unbroken continuity of management even during the Soviet era. Finally, our results show some patterns common to several countries and to the veterinary medicine promoted during the time of the Soviet Union. However, the Soviet Union and its centralized animal breeding system, resulted in a decline of ethnoveterinary knowledge as highly specialized veterinary doctors worked in almost every village. Future research should examine the complex networks of sources from where farmers derive their ethnoveterinary knowledge.

## Introduction

In many societies, livestock significantly contribute to human food security by providing several important food products, other valuable goods (e.g., wool, leather, and fat), agricultural inputs (e.g., manure), and services (e.g., transport, plowing). However, over the last century in the European context, animal production has been transformed by dynamics of centralization and decentralization (1, 2). These phenomena have also modified the associated veterinary knowledge and practices. While industrialized areas of Western Europe have increasingly shifted to highly technological animal breeding, in several rural communities of Europe, especially in mountainous regions, circum-Mediterranean areas, and post-Soviet contexts, livestock maintain their historical role in the livelihoods of peasants and they have been considered truly part of the family realm (3).

Animal breeding involves maintaining animal health and welfare. Scholars have found that the knowledge related to healing and ensuring the welfare of domestic animals has been largely abandoned in industrialized areas of Europe (3), while it appears to be relatively alive in rural contexts of the Mediterranean (4) and in some areas of Eastern Europe, e.g., Belarus (5–7). In the Mediterranean region, folk veterinary knowledge has been partially preserved due to geographical isolation and distance from veterinary services, yet it is rapidly declining and being replaced by modern livestock farming technologies and administrative veterinary controls (4). In several areas of Eastern Europe, a similar trend may have its roots in the political events that occurred during the time of the Soviet Union and after its collapse. Indeed, during most of the Soviet period, kolkhozes (collective farms) for animal (and crop) production were implemented, and each household could own only a small number of livestock (e.g., chickens, pigs, cows, sheep, goats) for subsistence (8). At the beginning of the 1990s, when the Soviet Union collapsed, political changes resulted in a profound transformation of agricultural production in Eastern Europe, with a concurrent decentralization and relocalization of veterinary knowledge production and implementation (9).

The use of plants for veterinary practices is well-studied in some regions of Eastern Europe. For instance, in Ukraine several scholars have investigated this topic [e.g., (10–13)], focusing on specific regions of the country (14, 15) or specific diseases (16, 17). However, most of the studies focusing on Eastern European ethnoveterinary medicine have been published in local languages. Indeed, in the international literature written in English, ethnoveterinary medicine is an underexplored field in Eastern Europe, but its preservation and implementation are increasingly considered a promising alternative for improving animal health and welfare. For instance, veterinary phytotherapy can find new applications in agroecological or organic agricultural practices, a fast-developing sector in Western Europe (18). In addition, ethnoveterinary knowledge can contribute to local biodiversity conservation (19, 20). Indeed, the health of an ecosystem and the livestock and people who inhabit it are strictly interdependent and need to be considered holistically (21).

Within this framework, our study aimed to document and compare current and past ethnoveterinary practices, and to identify trajectories in ethnoveterinary knowledge in rural borderland areas of eight countries from northern and southern Eastern Europe, namely Finland, Russia, Estonia, Lithuania, Poland, Belarus, Ukraine, and Romania. We further discuss what factors may have contributed to the persistence/erosion of ethnoveterinary knowledge in Eastern Europe.

## Materials and Methods

### Study Area

In the summers of 2018 and 2019, we conducted semi-structured interviews in four regions in eight countries that are home to nine main ethnolinguistic groups (Table 1).

**Table 1:** Main characteristics of the study area.

**Region**	**Country**	**Main groups**	**Languages**	**Number of SSI**	**Dominant landscape**
Karelia	Finland	Finns, Karelians	Finnish, Karelian	71	Plains covered by forests and lakes
	Russia	Russians, Karelians	Russian, Karelian	61	Plains covered by forests and lakes
Setomaa	Estonia	Estonians, Setos	Estonian, Seto	78	Mixed forests, fields, and marshy areas
	Russia	Russians, Setos	Russian, Seto	46	Plains covered by pastures and forests
Dzukija	Belarus	Lithuanians	Lithuanian, Belarusian	33	Plains covered by pastures
	Lithuania	Lithuanians	Lithuanian	30	Plains covered by pastures
	Poland	Lithuanians	Lithuanian	32	Mild hills covered by pastures
Bukovina	Ukraine	Hutsuls, Romanians	Ukrainian, Romanian, Russian	65	Mountain and hilly areas covered by forest and pastures
	Romania	Hutsuls, Romanians	Romanian	60	Mountain/Forest and pastures

Bukovina is a historical region of the Austro-Hungarian Empire that has been split between Romania and Ukraine since 1940. It is inhabited by several ethnic groups including Jews, Ukrainians, Poles, Romanians, and Hutsuls. Bukovina is partially occupied by the North-Eastern Carpathians which reach an altitude of 1,651 m a.s.l. We conducted our research among Hutsuls living in the Carpathian villages of the upper Suceava Valley in Romania and Putyla Rayon in Ukraine and among Romanians living in the pre-Carpathian hills of Straja (Romania) and Storozhenets and Glybotskyi district in Ukraine. Both in Ukraine and in Romania, most of the interviewees rely on family farming.

Dzukija is a historical and cultural region located in the borderlands of Poland, Lithuania, and Belarus. This border area has long been a crossroads for trading routes and has been subject to a series of changes in national status. It is now mainly inhabited by Lithuanians and Poles. It is characterized by plain and hilly rural areas and is currently experiencing a remarkable rural emigration. Soils throughout the studied region are sandy and of little agricultural value. We conducted interviews among Lithuanians living in several villages of Augustów and Sejny counties of Podlaskie Voivodeship (Poland), Šalčininkai district of Vilnius County (Lithuania), and Hrodna, Voranava, and Ašmiany districts of Hrodna Region (Belarus).

Karelia is a historical region currently divided between Finland and the Russia. Agricultural lands occupy only a small percentage of its territory, which is mainly covered by forests, a crucial resource for the local economy (22, 23). In Russian Karelia, during the last century, significant areas of meadow have appeared in place of abandoned arable land, but due to the cessation of grazing and haymaking, field degradation occurs which results in tree overgrowth and the spreading of shrub vegetation (24). In Finnish Karelia, forestland has been maintained over the last few decades through forestry intensification (25). On the Russian side, fieldwork was carried out in Petrozavodsk, Zaozer'e, Lekhnavolok, Novaya Vilga, Priazha, Essoila, Korza, Rubchoila, Siamozero, and Kalevala. On the Finnish side, the interviews were conducted for the most part in North Karelia (Joensuu, Lieksa, Nurmes, Valtimo, Ylä-Valtimo, Puukari, Rasimäki, Varpasenkylä, Viensuu, Viinijärvi, Sotkuma), while a few were conducted in Helsinki.

Setomaa is a region located at the Estonian-Russian border inhabited by speakers of the Seto and Russian languages. After WWII, the largest part of the formerly united historical Setomaa was incorporated into the Russian Soviet Federative Socialist Republic and remained there even after Estonia regained independence. Since the twentieth century, rapid alterations to the environment (like climate change and changes in the landscape due to the abandonment of agricultural activities) have led to the disappearance of some native plants (26) and the primary sector is continuously declining (27). Interviews were conducted in the villages of Pechorsky District of Pskov Oblast in the Russia and in Setomaa, Võrumaa-, and Tartumaa in Estonia. On the Russian side, most of the interviewees were retirees. During their lives, they worked in various positions in sovkhozes (state farms)–dairymaid, crop specialist, accountant, and head of the land plot. We also spoke to two zootechnicians–one has changed jobs since then while the other is still practicing.

### Field Study

**Figure 1:**
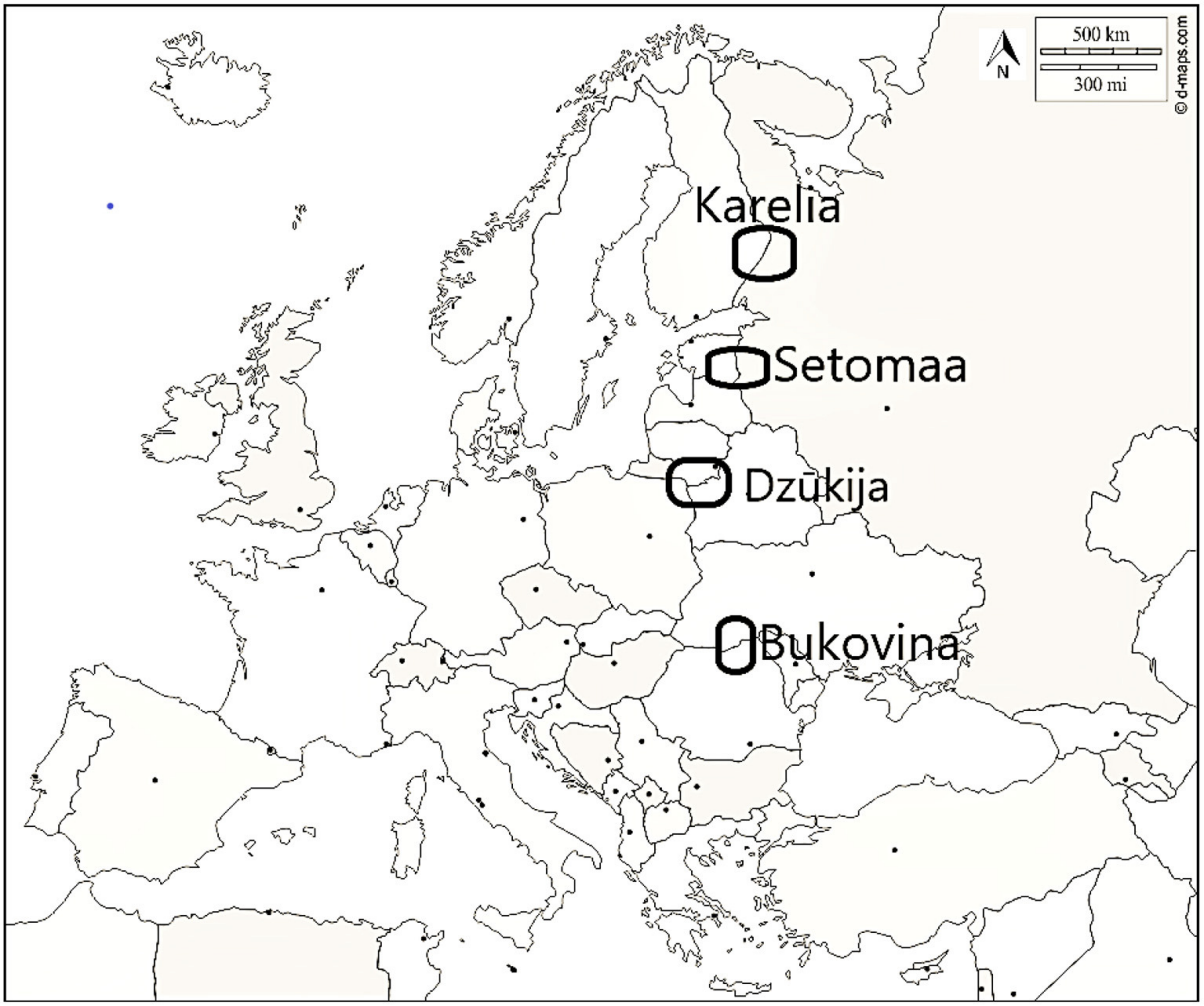
Research sites.

**Figure 2:**
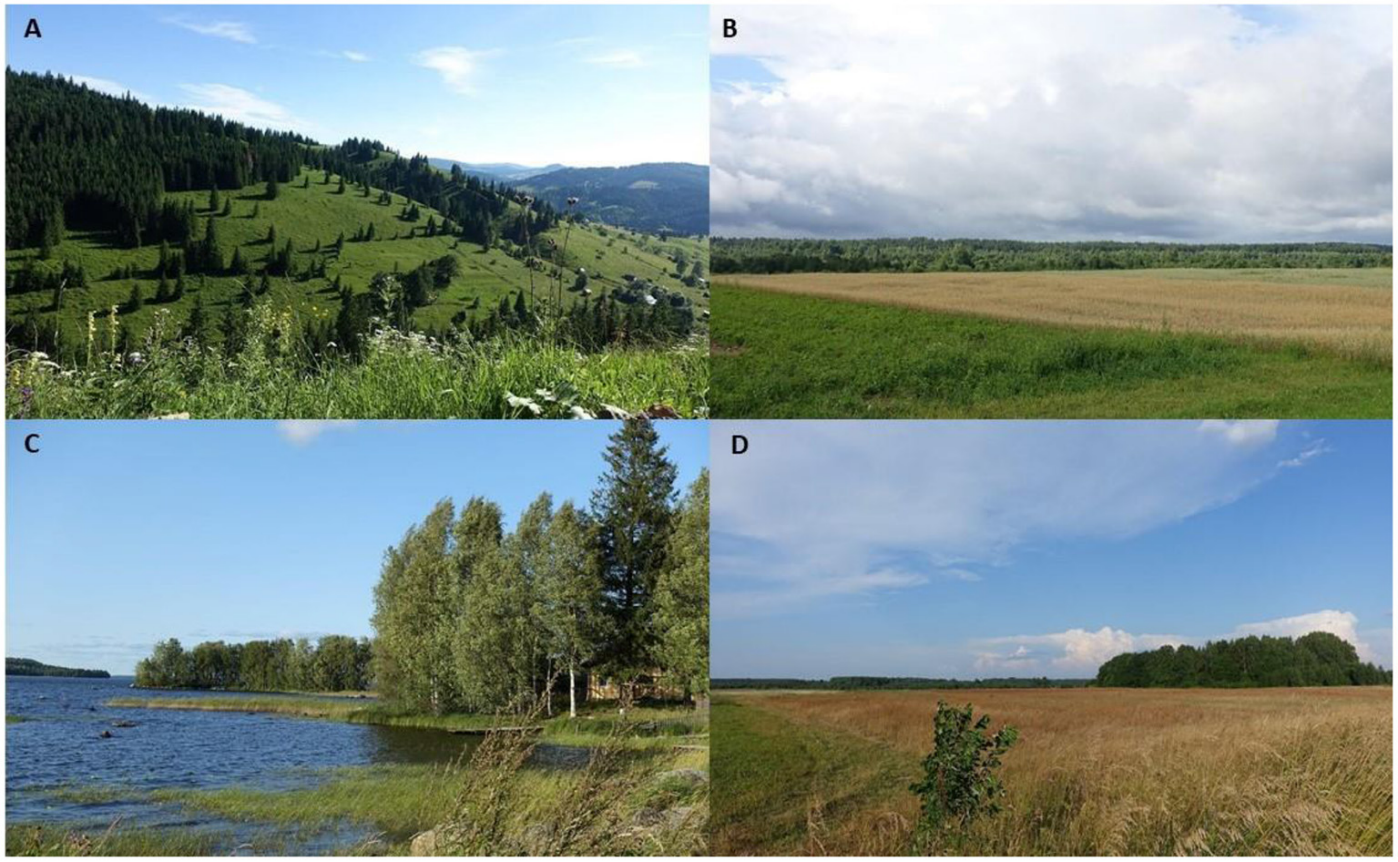
Landscapes of **(A)** Bukovina, Izvoarele Sucevei (Romania), summer 2019 (credit: Nataliya Stryamets); **(B)** Dzukija, Tabariškes (Lithuania), summer 2018 (credit: Julia Prakofjewa); **(C)** Karelia, Essoila (Russia), summer 2018 (credit: Valeria Kolosova); **(D)** Setomaa Panikovichi (Russia), summer 2018 (credit: Olga Belichenko).

The research was part of a wider study, namely the ERC-funded DiGe project, aiming to understand the mechanisms of change in ethnobotanical knowledge that occur among cross-border minorities when a dominant group tries to modify this knowledge. The four regions were selected in order to give an overall comparative picture of the current and past uses of plant-based ethnoveterinary remedies in Eastern Europe (Figures 1, 2). We conducted 476 semi-structured interviews among rural people conveniently selected while they were walking down the street, sitting on public benches (in the regions in which this was common), or working in their gardens. Our aim was to understand the existence and dynamics of ethnoveterinary knowledge among the non-specialist population living in the studied rural regions. We interviewed people mainly in one-to-one interviews, although on some occasions interviews involved other members of the household as well.

In Finnish Karelia, interviewees were also identified in advance through various social networks as it was not always possible to approach them directly. We consider this sample robust given the fact that saturation was reached after about 10–15 interviews per country.

Before each interview, prior informed consent was obtained following the Code of Ethics of the International Society of Ethnobiology (28). Upon consent, questions on ethnoveterinary medicine were asked in the context of documenting the whole plant use-system using the same methodology in all the countries. The questions were directed toward the healing of the livestock and other animals in the households using plant-based remedies. The general question on the presence of animals in the household was followed by questions regarding the healing of every animal mentioned by the interviewee and on the ways in which these plant-based products improved the quality of animal products (e.g., milk, meat) both in the past and in the present. We consider quality that which, in different ways and based on interviewees' perceptions, improves, enhances, and intensifies the characteristics (e.g., in terms of taste, smell, nutritional properties, etc.) of milk and meat. We allowed interviewees to freely talk about the topic, asking follow-up questions when needed. When a wild plant was mentioned, we asked the interviewee to show it to us if possible. Collected voucher specimens are stored in the herbarium of Ca' Foscari University of Venice (UVV) for countries of the European Union (Finland [bearing numbers KAR02–KAR012; KARDR10 and KARDR24], Estonia [bearing numbers SE001–SE135], Lithuania [bearing numbers DZULT01–DZULT126, and DDZULT01–DDZULT45], Poland [bearing numbers DZUPL001–DZUPL107 and DDZUPL01–DDZUPL39], Romania [bearing numbers SB003–SB096]), while the specimens collected outside of the EU are deposited in Roztochya Nature Reserve for Ukraine [bearing numbers NB001–NB085] and in the Komarov Botanical Institute, Russian Academy of Sciences, Saint Petersburg for the Russia [bearing numbers LE 01063392–LE 01063946 (http://en.herbariumle.ru/)].

When possible, interviews were recorded upon the interviewee's approval and transcribed in the local language; in the few cases when recording was refused, we took notes. Later, we entered this information in English into an Excel spreadsheets organized as detailed use reports (DUR) of plant-based remedies, where each row contained the country and ethnic community of the interview, its code, the scientific name of the plant, its local name, the part used, when it was used, the mode of preparation, and its use. Botanical taxa were classified using World Flora Online (2021). The botanical families were classified according to the Angiosperm Phylogeny Website (29).

The research protocol was approved by the Ethics Committee of Ca' Foscari University of Venice.

## Results

**Figure 3:**
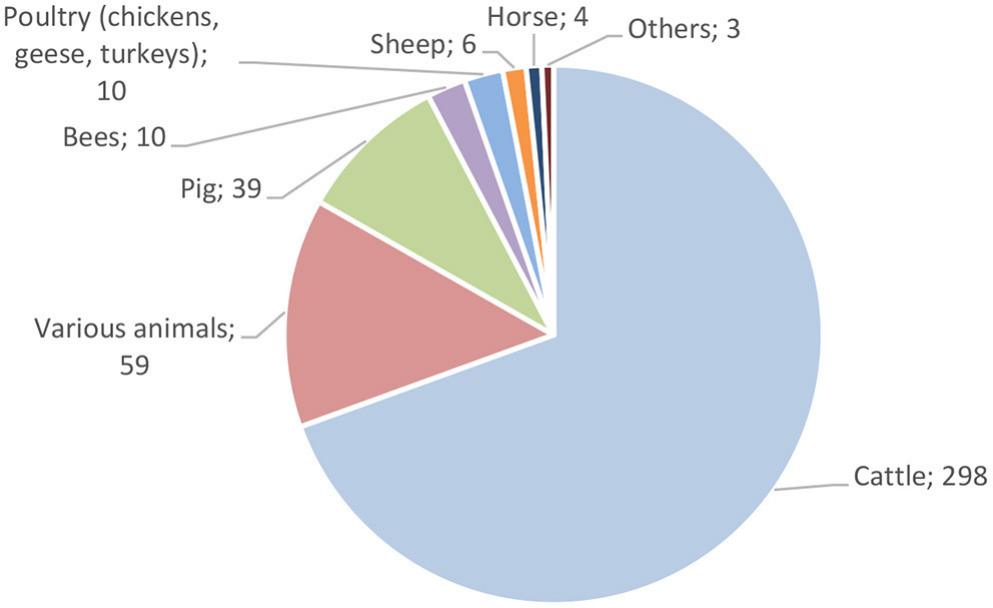
Distribution of ethnoveterinary use reports by animal category.

In the four regions where this study was conducted, we recorded the use of 94 plant taxa, 67 of which were wild and 24 were cultivated, from 189 interviewees. We documented 452 use reports, 19 of which were related to the improvement of the quality or quantity of meat and milk, while the other 428 involved ethnoveterinary practices for treating 10 domestic animal taxa. Out of the 476 interviews we conducted in the four study regions, 189 reported ethnoveterinary uses. Below, we focus first on ethnoveterinary knowledge related to cattle as about 70% of the use reports concerned cattle illnesses (Figure 3).

### The Importance of Cattle

Cattle were the most mentioned target of ethnoveterinary treatments across all the study areas. We recorded the use of 55 plants belonging to 25 families (Supplementary Tables 1, 2) for this purpose, 35% of them were cultivated and 65% were wild.

Only four species were reported in five or more countries (*Artemisia absinthium, Hypericum* spp., *Linum usitatissimum, Quercus robur*).

**Figure 4:**
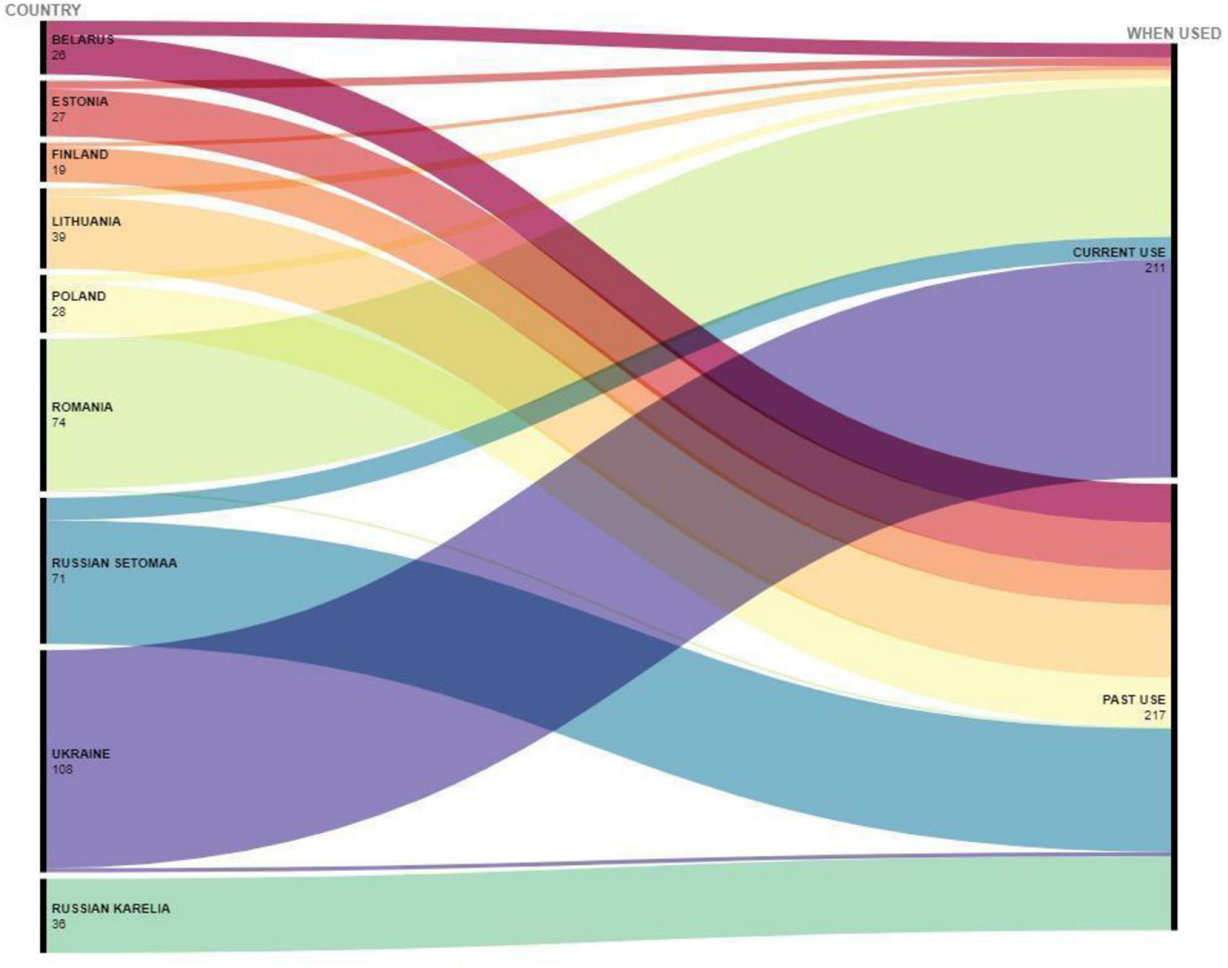
Alluvial graph of the number of ethnoveterinary remedies (colored stripes), country (on the left), and time of use (on the right).

The majority (61%) of the DUR were currently in use, while 39% referred to past uses. However, excluding the Bukovinian Carpathian area (our Ukrainian and Romanian case studies), the proportion of currently used DUR drops to 12%, while the remaining 88% refers to past ethnoveterinary uses. Indeed, as illustrated in Figure 4, current uses were widely reported in Romania and Ukraine and to a lesser extent in Russian Setomaa, Poland, and Belarus. Karelians, Estonian Setos, and Lithuanians referred only or mainly to past uses.

**Figure 5:**
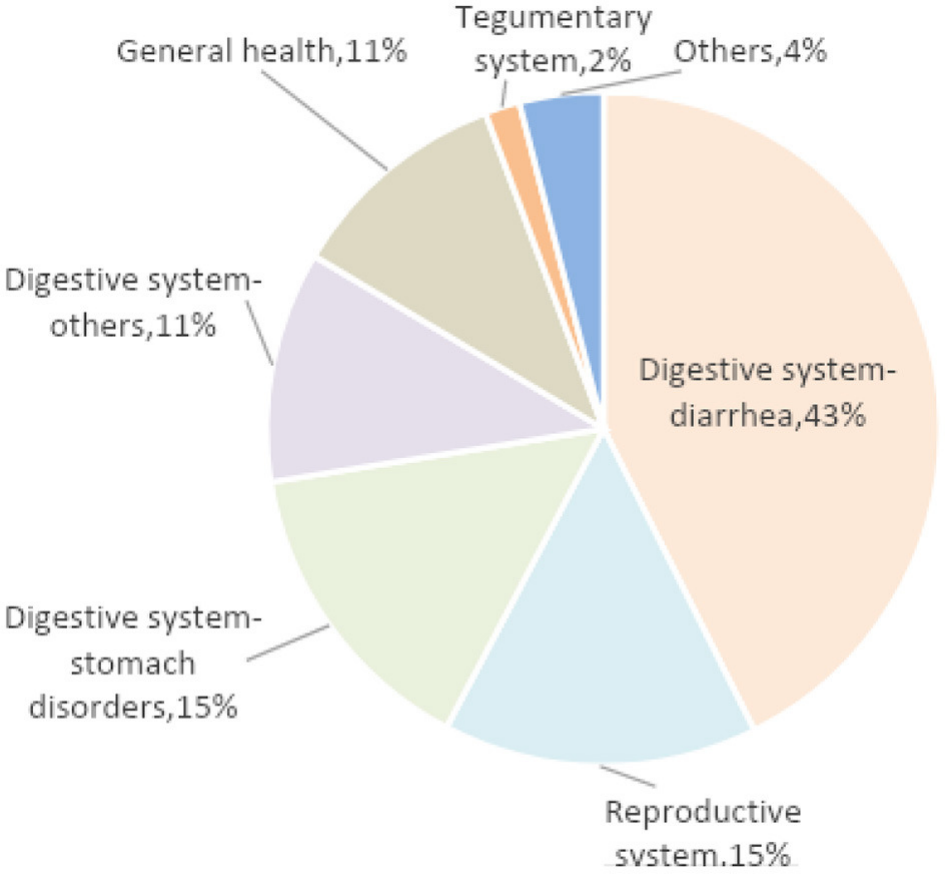
Distribution of detailed use reports (DUR) related to cattle ethnoveterinary remedies.

The digestive and reproductive systems were the most common targets of ethnoveterinary remedies (Figure 5). For the digestive system, diarrhea was the most reported ailment followed by stomach illnesses and other issues such as intestinal gas, rumination problems, and abdominal pain. For treating diarrhea, the most commonly used plants were *Rumex* spp. (reported in four countries across three regions), *Hypericum* spp. (reported in five countries across all four regions), and *Quercus robur* (reported in five countries across all four regions). In regard to the reproductive system, the most common issues involved calving and the use of postpartum supplements. The most utilized plant for treating the reproductive system was *Linum usitatissimum*, mentioned in four countries and three regions. Among the listed plants some (e.g., *Atropa belladonna, Cannabis sativa, Hypericum* spp.) could potentially have negative effects on animals. While no interviewee explicitly mentioned possible adverse side-effects, they were not assessed, being out of the scope of this article.

### Ethnoveterinary Remedies Related to Other Livestock

Excluding cattle, we documented 130 DURs related to 44 plant taxa belonging to 28 botanical families that were used for treating nine animal taxa, including pigs, honeybees, sheep, turkeys, chickens, geese, horses, dogs, and cats. Only three species were found to be used in four countries: *Picea abies* (three regions), *Quercus robur* (three regions), and *Urtica dioica* (two regions). The livestock most commonly treated with ethnoveterinary remedies were pigs (39 DURs), which were mainly mentioned in Russian Karelia and Russian Setomaa. As in cattle, the most widely treated illness was diarrhea, primarily in pigs. Also, five plant taxa (above all *Urtica dioica*) were used, especially in Dzukija, as feed supplements (13 DURs).

### Plants to Improve Animal Product Quality

In addition to ethnoveterinary remedies, we also recorded the use of 19 DURs referring to ten plant taxa for improving the quality of milk, meat, and in one case pig bristles (Table 2). More than half of the uses (11) were reported in Bukovina, the rest in Lithuania and Russian Setomaa. None were reported in Karelia. The most common plant was *Urtica dioica*, which was reported by six people in Bukovina for improving milk quality and by one interviewee in Lithuania for improving pork meat quality.

**Table 2:** Plant taxa used for improving the technological quality of some animal products.

**Latin name and family**	**Local name**	**Part used**	**Preparation**	**Use**	Use reports (^*^past)
*Arctium tomentosum* Mill. (*Asteraceae*) [W]	Лопух (RS)	Leaves	Fresh	Improving cow milk quality	RS
*Chelidonium majus* L. (Papaveraceae) [W]	Czystaciel (LT); чистотіл (UA)	Aerial parts	Fresh; Infusion	Improving cow milk quality	2 UA; LT^*^
*Chenopodium album* L. (*Amaranthaceae*) [W]	Balanda (BL)	Aerial part	Fresh	Improving pork meat quality	LT^*^
*Linum catharticum* L. (*Linaceae*) [C]	Linučiai, linuciai (LT)	Seeds	Infusion	Improving cow milk quality (increase fat in the milk)	LT^*^
*Linum usitatissimum* L. (*Linaceae*) [C]	Лён (RS)	Seeds	Fresh	Improving pig bristle quality	RS^*^
*Rumex confertus* Willd. (*Polygonaceae*) [W]	Шіва (UA)	Leaves	Fresh	Improving sheep milk and wool quality	UA
*Salix* spp. (*Salicaceae*) [W]	Paju (FI)	twigs and leaves	Fresh or dried	Improving cow milk quality	FI^*^
*Stellaria media* (L.) Vill. (*Caryophillaceae*) [W]	Žliuge (LT)	Aerial parts	Fresh	Improving pork meat quality	LT^*^
*Trifolium* spp. (*Leguminosae*) [W]	Trifoi (UA)	Aerial parts	Fresh	Improving cow milk quality	UA
*Urtica dioica* L. (*Urticaceae*) [W]	Dilgele (LT); Urzica (RO); кропива (UA)	Aerial parts	Infusion	Improving cow milk quality	4 RO; 2 UA
			Fresh	Improving pork meat quality	LT^*^

*W, Wild species; C, Cultivated species; LT, Lithuania; RO, Romania; RS, Russian Setomaa; UA, Ukraine*.

* *No longer in use, number of use reports (UR) is indicated if more than 1*.

### Ethnoveterinary Knowledge Dynamics

Out of the 476 interviews we conducted in the four study regions, 189 interviewees reported ethnoveterinary uses (Table 3). Specifically, Bukovina had the highest percentage of people who mentioned ethnoveterinary remedies (58%), followed by Dzukija (44%) and Setomaa (41%). Karelia was the region where less ethnoveterinary knowledge was reported (only 21% of respondents could mention at least one remedy). Bukovina was also the region with the highest rate of DUR per person (2.5), followed by Dzukija (2.2). When examining the data from a cross-border perspective, we can observe that bordering countries in Bukovina and Karelia reported similar percentages of interviewees mentioning ethnoveterinary remedies and DUR referring to the past. However, in Dzukija, especially in Poland and Lithuania, a higher percentage (86 and 90%, respectively), of past uses were mentioned compared to Belarus (73%). Similarly, in Setomaa the percentage of interviewees who could mention at least one ethnoveterinary remedy was 23% in Estonia, while it was more than double that (59%) in Russian Setomaa.

**Table 3:** Ethnoveterinary knowledge by region.

	**DUR (*n*)**	**DUR referring to past uses**	**EV respondents (*n*)**	**Total respondents (*n*)**	**% of interviewees who mentioned EV remedies**	**% of DUR referring to past uses**	**DUR per interviewee (*n*)**
Romania	74	1	34	60	57	1	2.2
Ukraine	108	2	38	65	58	2	2.8
**Total Bukovina**	**182**	**3**	**72**	**125**	**58**	**2**	**2.5**
Belarus	26	19	15	33	45	73	1.7
Lithuania	39	35	15	30	50	90	2.6
Poland	28	24	12	32	38	86	2.3
**Total Dzukija**	**93**	**78**	**42**	**95**	**44**	**83**	**2.2**
Finland	20	18	15	71	21	90	1.3
Russian Karelia	36	36	15	61	25	10	2.4
**Total Karelia**	**56**	**54**	**30**	**132**	**23**	**95**	**1.9**
Estonia	27	23	18	78	23	85	1.5
Russian Setomaa	71	60	27	46	59	85	2.6
**Total Setomaa**	**98**	**83**	**45**	**124**	**41**	**85**	**2.0**

### “I Call the Doctor!” and Other Attitudes Toward Ethnoveterinary Practices

We recorded different attitudes regarding the use of plants for treating animals (Table 4). Interviewees often expressed a dichotomy between official veterinary services and plant-based solutions. For instance, when asked about their actions in the case of animal illness, several interviewees said, “Normally we call the veterinary doctor” (Romanian Hutsul man born in 1951) and “I call the vet and he gives me something but I do not know what” (Romanian Hutsul man born in 1934). In Ukraine, some interviewees specified that “Now we go to the doctor” (Ukrainian Romanian woman born in 1939) or “For this [mastitis], now we need to call the doctor” (Ukrainian Romanian woman born in 1983), underlining that this was not the case in the past.

**Table 4:** Prevalent attitudes toward ethnoveterinary remedies.

**Prevalent attitudes toward ethnoveterinary remedies**	**Countries in which the concept was mentioned**
(Now) we call the veterinary doctor	Romania, Ukraine, Belarus, Russian Karelia, Russian Setomaa, Estonia, Lithuania, Poland, Finland
Better to use drugs, they are more reliable	Belarus, Poland, Lithuania
Better to use drugs, they are faster	Russian Setomaa, Russian Karelia
Better to use herbs, they are more effective	Romania
In the old days, animal healers knew incantations	Russian Karelia, Russian Setomaa, Belarus, Lithuania
And if we can, we cure the cattle ourselves	Russian Karelia, Romania

In the Dzukija region, older respondents reported the loss of ethnoveterinary knowledge as a reason for turning to official veterinary medicine: “There are no more people who know the right herbs and remedies. Now there are veterinarians. If someone needs something, they turn to veterinarians” (Belarusian Lithuanian woman born in 1946). Informants from both the Belarusian and Lithuanian sides of Dzukija noted that state-sanctioned veterinary medicine appeared with the advent of the Soviet state and the formation of kolkhozes [~1950]. On the Polish side, older Lithuanian interviewees mentioned that they mainly healed animals with drugs (Polish Lithuanian woman born in 1929).

At the same time, although the older generation of interviewees living on the Belarusian and Lithuanian sides of the Dzukija region is characterized by a lack of trust in official medicine, the middle and younger generations prefer medicines over medicinal herbs. For example, a middle-aged female respondent noted that it is better to treat cows with drugs, not herbs. For her, using chemicals is a more reliable option (Lithuanian woman living in Belarus woman born in 1954).

Among the respondents who worked on kolkhoz between the 1950s and 1990s, some noted that they used home remedies for the kolkhoz animals: “I worked on the farm, raised calves, and none of them died. I made bottles for them at home. There were pałyn (*Artemisia absinthium*), čystacieł (*Chelidonium majus*). And I carried these bottles to the kolkhoz” (Lithuanian woman living in Belarus woman born in 1941).

In Russian Karelia and Setomaa, the decision to call a veterinary service (instead of treating livestock using plant-based remedies) was sometimes perceived as a matter of time, as one veterinary doctor mentioned, “You know, when I started working, there were already antibiotics […]. But even at the beginning of my work [in the late 1970s], in fact, they did not pay much attention to folk remedies. Well, maybe because this is a longer path to recovery, whereas one needs the result immediately. After all, they use more modern ones here” (Russian Seto woman born in 1939). This was also confirmed by a retired kindergarten nanny: “And as with pills, you call a doctor, because it is fast for calves, yeah, for calves. Quicker with them, because herbs are too slow” (Russian woman, born in 1962, living in Karelia). Conversely, in Romania, some older interviewees were skeptical about official veterinary medicine: “Teas of *Hypericum, Equisetum, Carum carvi*, dried bread, and the grains of *Coriandrum sativum* are better than furazolidonul [a vet medicament]” (Romanian woman, born in 1948, living in Romania).

In the Soviet Union, it was not difficult to receive veterinary consultations (“The vet lived right here. They [vets] came on foot, came [by bus or truck], some on horseback,” said a Russian Seto woman born in 1956). However, some interviewees also reported that “there were specialists, but at home, you were mostly on our own” (Russian Karelian man born in 1950). Contrastingly, another interviewee stated, “Cattle were not treated at home, only by a veterinarian. In the old days, there were very good veterinarians. Only the veterinarian treated animals” (Russian Karelian man born in 1929). Indeed, during Soviet times there was a veterinary doctor working in every large village with kolkhoz who helped locals to treat cattle. An Estonian Seto woman born in 1938 narrated: “Animals had flax seeds after calving, but otherwise, if something was wrong, we had to call the vet.”

In Finnish Karelia, an interviewee claimed that in the 1950s, “Veterinarians were not used as the nearest veterinarian was 35 km away. There was someone among the people, who was familiar with these problems, and he/she was called only if there was some illness and you were unsure of what you should do” (Finnish woman, born in 1978, living in Finland).

### Human-Livestock Connectedness in Eastern Europe

Our interviewees from the four regions often mentioned the deep connection they have or had with their livestock. For instance, in Russian Karelia, an Ingrian-Russian woman (born in 1954) mentioned, “We had goats, sheep, yes. well, at that time it was kind of necessary, because living was not very. how to say?–easy. Therefore, we kept them,” stressing the role livestock played in guaranteeing food security.

Also, a Russian woman, born in 1966, living in Karelia reported using human food for feeding animals: “They just subsidized bread very, very much. […] My Karelian grandmother fed all her cattle with bread. It was cheaper than growing potatoes and buying grain from a sovkhoz. And my dad, having a car, every day brought her two loaves, four loaves, of bread. It was a strange idea of our household, but bread was subsidized, and the rest was not.”

In Romania, a Hutsul interviewee (Romanian Hutsul woman born in 1961) clearly explained the nexus between “eating in a healthy way and livestock breeding. When asked about any possible product she could give animals to produce more milk she exclaimed, “Ah, no, I do not give them anything. Well, to be precise, sometimes a bit of bran, when animals come in at night and in the morning when milking, but otherwise, grass. Here we eat healthily.” It is worth noting how “we” includes both humans and animals, as a whole, and therefore if animals eat healthily humans will as well.

In the Dzukija region, however, among the villagers, there is often a reasonably practical approach: “We did not contact anyone. If an animal lives, it lives. Nothing was treated. As the old then died” (Lithuanian woman living in Belarus woman born in 1939). We recorded a profound respect for livestock. Many respondents said that, for example, a cow is a very clever animal that knows what to eat and what not to eat. At the same time, the respondents remembered many instances of using herbal remedies in various rituals related to the prevention of diseases in livestock. In particular, the tradition of blessing animals with a palm bouquet has survived to this day: “And also animals were smoked with that Easter Palm. Yes, if they got sick then they did it” (Lithuanian woman living in Lithuania born in 1938). Setos used *Salix* sp. twigs consecrated on Palm Sunday. They placed these twigs on the barn door to protect animals. Both Seto and Russian people recalled drawing a black cross with charcoal above the barn door, on baptism day, to protect animals.

It should also be noted that a large number of our respondents' recollections concerned the use of various plants for the treatment and prevention of culture-bound illnesses in domestic animals, such as fright and the evil eye: “For fright, there is uročnikas [*Gymnocarpium dryopteris*] […] which is good for the evil eye as well. […] Especially for animals, we used to collect it, if someone casts the evil eye, you smoke it, and it was a medicine” (Lithuanian woman living in Lithuania born in 1939).

### Perceived Changes in Livestock Keeping

Among the changes in livestock keeping mentioned by interviewees who lived during Soviet times, the crucial role of cows was reported: “When collectivization took place, only one cow was left for the family. Whether it was one person, or seven people, still only one cow was left for the family. And they also handed over meat, milk, and eggs [to the procurement system]. It was a difficult period then” (Russian woman, born in 1940, living in Setomaa). This was also confirmed by an elder Finnish Karelian woman who remembers that whipped cream from the only cow remaining was an important resource: “I remember, in the 1970s, when it was decided that we would be left with one cow. At that time, cream was skimmed from milk and whipped as whipped cream.” Another participant commented, “It was not considered a farm if there was no cow” (Russian Karelian man born in 1928). In addition, a retired Karelian seamstress (born in 1954) living in the Russia mentioned that cattle disappeared because “the mowing was very bad, and it was necessary first to give hay to the sovkhoz, and therefore my parents got rid of the cows.” This issue of the lack of hay was also mentioned by a Karelian forestry specialist: “It was torture to keep a cow. When I was in elementary school, my father had a MAZ lorry. Mowing was not allowed then, and they drove in the direction of Interposiolok to steal hay; they mowed on the side of the road and returned with it at night–either to the neighbors or to our own farm. They helped each other, dried. they dried hay, we dried hay, and that's all. so. and lived” (Russian Karelian woman born in 1948).

Among other more recent changes, a Seto interviewee claimed, “And now you can list the cow in the Red Book, only a few cows remain. So, there are no animals to treat actually” (Russian Seto man born in 1943). An Estonian Seto woman, born in 1938, commented, “I had three cows, and for a short time also four. When the Estonian state started, it destroyed all the cows, no one needed milk anymore. Milk churn stands were lost.” A younger Estonian Seto man (born in 1953) explained his point of view, referring to the current availability of state jobs: “There were those “state” works [e.g., kolkhoz and sovkhoz], and, on top of them, people had their own animals and farmland. But have you seen that now no one raises animals like cows or pigs at all? Pigs can't be kept anymore, because that bastard plague (African swine fever) is here.” In Ukraine, an older Romanian woman explained, “I do not have a cow now, but I had one. I have had it for many years, but now I am old, weak and I really cannot keep it.”

A couple of Romanian Hutsuls reflected upon our question regarding agricultural changes in the last few decades: “Agriculture has changed since our youth. It has changed a lot […]. Now they make much more hay, but the grass lasts, which in the past did not happen because everyone here had livestock, much more than now since there are no animals, because milk is paid almost nothing, and so why keep a cow if the milk… [is paid nothing]” (Romanian Hutsul man and woman born in 1934 and 1939, respectively). However, they also proudly stated, “Milk here is natural, from flowers, cows graze on flowers and also eat hay. The milk is fatty, it is good.” Finally, a Romanian teacher living in Romania claimed, “I have had livestock, but my children have left, parents have passed away, and working and caring for animals is hard, so I gave it up,” and then she continued counting the number of livestock in the village: “For instance, my grandparents had a lot of livestock, sheep and cows, and in summer they used to go to the mountain pastures, but now I think that in the entire village only five people go to the mountains. Also, the animals are few: we had about 50 animals, the same number as our neighbors, but now, in all the area, there are barely 10.”

In the Dzukija region, the respondents noted that cows grazed everywhere in the village in the recent past [1980–1990s]. “And only this year locals killed the last cow” (Lithuanian woman living in Belarus woman born in 1939). Now, mostly older people keep at the most chickens, although, in comparison with the past, they had a rather large farm. Thus, a respondent from the Belarusian side of Dzukija noted that in her youth, she and her husband kept three cows, three pigs, a horse, a lot of chickens, geese, and sheep, and now she only has three chickens (Lithuanian woman living in Belarus woman born in 1946).

The most evident difference in livestock keeping was in Poland, where the Lithuanian minority are larger-scale farmers. They still keep many animals and sell the milk to the state. In Communist times, farming in Poland was nationalized only to a small degree. Just a small proportion of food production was state based, while the majority remained in private hands. This also meant that private farming in Poland was highly fragmented, as the state did not encourage mergers and land acquisition.

## Discussion

**Figure 6:**
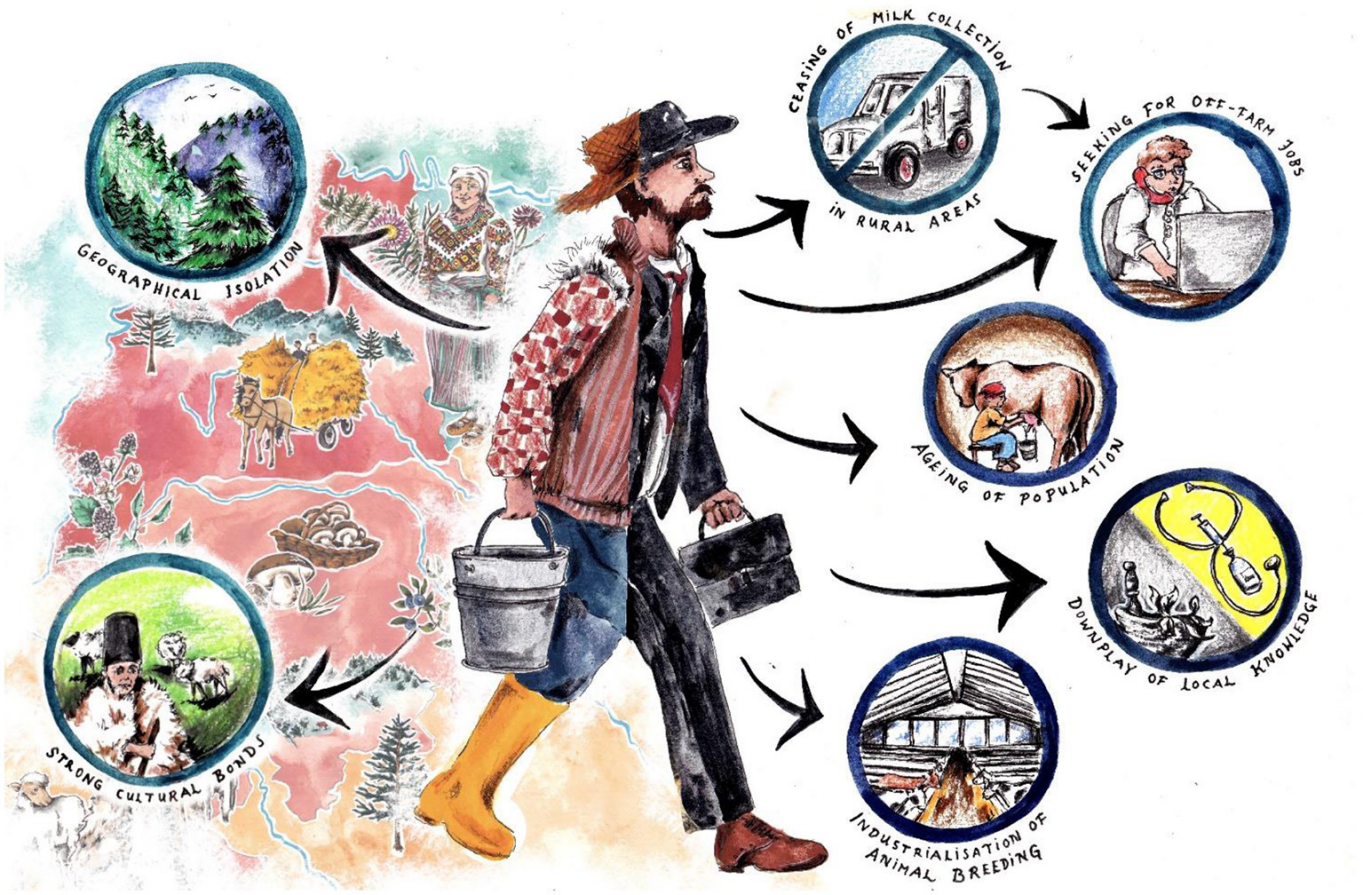
Possible drivers of the persistence of ethnoveterinary knowledge in Bukovina (left) compared to possible drivers of ethnoveterinary knowledge erosion in Setomaa, Dzukija, and Karelia (Credits: Johanna Lohrengel).

Our results showed that the four study regions located in Eastern Europe do not present similar ethnoveterinary knowledge trajectories. Bukovinian Romanians and Hutsuls appear to hold a living reservoir of ethnoveterinary knowledge, unlike the other regions as summarized in Figure 6. However, despite differences in the richness of ethnoveterinary knowledge, cattle were the livestock most commonly treated with herbal remedies in all four regions. Setomaa (especially Estonian Setomaa) and Dzukija showed an erosion of ethnoveterinary knowledge with many uses reported in the past but no longer in use.

### Bukovinian Carpathians as a Reservoir of Ethnoveterinary Knowledge

The current richness of ethnoveterinary knowledge reported in Bukovina could have been developed and maintained through its peculiar geographical location. Unlike the other study areas of this research, our fieldwork in Bukovina was carried out in mountainous and peri-mountainous areas. For centuries, the (Bukovinian) Carpathian Mountains have served pastoralists and specifically transhumant herders (30). In particular Hutsuls, one of the studied ethnic groups, are well-known for their centuries-long expertise in mountain pastoralism (31). We observed that several Hutsuls still use their *polonyna* (summer pastures) or send their animals out with other shepherds. However, long-term (pacific) coexistence of Hutsuls with other ethnic groups, could have facilitated the sharing of their knowledge of animal breeding (especially ethnoveterinary practices) among Romanians living in neighboring pre-Carpathian areas. Therefore, the mountainous nature of the area could be a key element in the resilience of ethnoveterinary knowledge, which has also been confirmed by the intrinsic relationship found between pastoralists and European mountains [e.g., (32, 33)] and possibly, as a result, the related ethnoveterinary knowledge (34). In a geographically remote and rather inaccessible territory, such as the Carpathian (and pre-Carpathian) territory of Bukovina, cattle play a crucial role in terms of food security and economic importance, especially in times of crisis. Still, at the present time, Hutsuls and Romanians rely heavily on animal husbandry based on small-scale family farms (35). While sheep are very important from a cultural perspective, especially among Hutsuls, the small proportion of sheep remedies mentioned may be due to several factors including their limited economic importance (it is cow, and not sheep, milk which provides income), and possibly to the fact that we conducted interviews in the summertime when sheep are not with their owners but in mountain areas with shepherds who might have special treatments for sheep which the owners do not know. Therefore, livestock husbandry, particularly cattle breeding, is a daily activity for both Hutsuls and Romanians living in Bukovina. This is specifically due to the importance of milk, which can provide a regular and reliable source of protein and/or cash income throughout the year, in a context where no other agricultural commodities can do this, as observed by Kitching (36). Indeed, milk, when processed, is a crucial element of the local diet and cuisine (30, 37). Among the dairy products which play an important cultural role, identifying Hutsuls as mountain pastoralists, the most salient is bryndza, a complex cheese produced in summer pastures, which has recently obtained Geographical Indication status when produced with sheep milk in the Rakhiv region [the first in Ukraine, (38)] and has been included in the Ark of Taste promoted by Slow Food (39). Other dairy products, including budz (a smoked cheese produced among Ukrainian Hutsuls) and (v)urda (a soft cheese), also contribute to transfer the centuries-long interaction with the Carpathians into the Hutsul foodscape.

Such everyday practices in animal breeding (milking and cheese-making) are still currently present and so is the veterinary knowledge connected to them. Indeed, as observed by Warchalska-Troll and Troll (40), Carpathian pastoralism was characterized by an unbroken continuity of management even during Soviet times. This could be a key for the resilience of the ethnoveterinary knowledge in the area. While several interviewees reported calling the veterinarian for serious issues, most Bukovinian interviewees reported treating minor ailments related to the digestive system and calf birthing themselves, using ingredients (such as plants) locally available. Often, people mentioned that “this is good for both people and cows,” revealing the tight nexus between their own well-being and that of their cattle. These thoughts may have been fostered by the habit, in several areas of the Soviet Union, to buy bread to feed livestock, a practice which became so popular that a decree banned this “misuse” of bread in 1957 (41). This is also reinforced by the observation of Kitching (36) for whom the smallholder production of livestock (and crops) during Soviet times (and right after) represents a “remarkable history of survival and productivity.” Indeed, in the 1990s, home-grown food also played a key role in the survival strategy of communities (42) in several post-Soviet contexts, including Northern and Southern Bukovina. Thus, livestock were somehow needed and cared for as valuable family members. The harsh times and this diffused sense of “extended family realm” reveal a strong connectedness to and a holistic vision of the ecosystem where people and livestock are equally important actors within a larger system.

### Possible Erosive Trajectories of Ethnoveterinary Knowledge in Karelia, Setomaa, and Dzukija

In Karelia, agriculture has been marginalized, especially in terms of the area where hay is still cut and the number of natural pastures, due to the declining number of cattle and the intensification of dairy farming (43–45). We can therefore hypothesize that our Karelian interviewees did not mention many ethnoveterinary remedies for three main reasons. First, due also in part to the climatic conditions, agriculture and animal breeding have often played a marginal role throughout Karelian history and therefore few livestock animals were kept, although their numbers have been sharply declining over the last century (46, 47). This was also confirmed by an interviewee who stated, “I ate my first chicken at 18 and a half years old. We always have elk and game. Why do we need artificial chickens?” (Karelian Russian woman born in 1968), underlining that wild food plays a major role in the Karelian diet. Second, Naumov et al. (48) reported that in Russian Karelia cattle breeding is currently highly subsidized and the subsidies may help pay for veterinary services that were well-developed during Soviet times (9), thus abandoning existing ethnoveterinary knowledge.

In Setomaa (especially Estonian Setomaa) and Dzukija, we recorded more uses, but mainly in the past. We can say that, in these territories, livestock were quite important in the recent past, but their importance has declined in the last few decades [e.g., (49) for Belarus and Poland; Kalle and Kass (50) for Estonia]. Our interviewees in Estonia still remember when the “one cow” restriction was lifted in the 1980s and everyone was allowed to keep as many animals as they were able to. The restrictions were lifted as private livestock farmers provided a significant portion of the country's milk. Collective farming system could no longer feed the people and so the economic model had to change. The main reason why cows are no longer kept in Estonia is that dairies stopped buying milk from small rural households during the transition to a market economy. At that time, keeping cows became no longer economically viable because there was nowhere to sell the milk.

The process of knowledge erosion may be occurring as a result of different socio-economic changes. First, in both regions, the aging of the rural population has contributed to the abandonment of small-scale livestock breeding (51–53). Second, the trend of “industrializing” livestock has reduced the number of farms and increased the number of animals per farm [we also observed this phenomenon in Finnish Karelia, and it has been found in the Mediterranean context as well, e.g., (54)]. This phenomenon gathered livestock farms in specialized districts and makes use of specialized “academic” knowledge, rather than any local veterinary knowledge. Third, in Setomaa, we observed that an increasing number of people are looking for off-farm jobs (e.g., government employment) which provide a monetary wage and are thus more appealing than farming activities. Such a change started in Estonia in the 1990s with the transition to a market economy, when the planned economy was abolished. In this type of shift, animal husbandry, which requires daily attention, is often the first activity to be abandoned, followed by labor-intensive crop production. Fourth, the policies concerning food production that have been implemented by the European Union in the last several decades may have impacted the system of livestock farming (55, 56). Finally, there may be a widespread tendency to downplay local knowledge by preferring “official veterinary” medicine, stressing the sanitary and economic perspective of animal husbandry. This phenomenon may be also fostered by the increased income and availability of official veterinary medicine. Our interviewees in Estonia explained that the main reason why it was necessary to call a veterinarian was that the bacterial content of the milk was measured every time milk was sold. If an animal had inflammation, no milk could be purchased, so the animal had to be cured quickly, with an antibiotic; yet one was not allowed to sell milk during treatment. Thus, people lost financially when they simply poured milk into the sewers, and one of the main reasons why the animals were not treated at home with their own resources was that there were strict hygiene requirements for the sale of milk and meat. In addition, today, sanitary requirements to prevent African swine fever have closed all small pig farms.

In addition, some local drivers of decline of ethnoveterinary knowledge can be identified. For instance, in Russian Setomaa, one possible driver could be related to the drastic political and economic changes in the region. Indeed, during Soviet times, animal husbandry accounted for a relevant proportion of food production by ruble value, while after 1991 livestock herds declined precipitously and the number of livestock raised by households continues to decline (57, 58). Also, according to our observations, in Belarus (and partially in Lithuania) a rapid decline in rural population size is one of the drivers of livestock husbandry abandonment and its associated knowledge. In most cases, during the autumn-winter period, the older generation of respondents goes to live with their children in nearby settlements. When we looked for the most senior residents, most of them were in the city under the care of children and usually returned home only in the spring and summer. Another possible factor that affects the evolution of ethnoveterinary knowledge in present-day Dzukija concerns political decisions related to restrictions on keeping domestic pigs and birds in the border region. This is linked with the implementation of EU and the Republic of Belarus regulations in 2014–2020 to limit the threat of African Swine Fever. These political decisions endanger traditional livestock practices by undermining the very core of their existence, and, accordingly, the practice of ethnoveterinary medicine.

### Possible Sources of Ethnoveterinary Knowledge

Our results show that ethnoveterinary knowledge partially overlaps in the selected regions, especially in countries formerly part of the Soviet Union. For instance, some taxa such as the cultivated *Linum usitatissimum* and the wild *Hypericum* spp., *Quercus robur, Alnus* spp, and *Rumex* spp. were reported across several countries. As five out of the eight studied countries were part of the Soviet Union, we compared our field data against the uses recommended in three popular veterinary medicine books published in 1919 (Gurin), 1988 (Rabinovich), and 2007 (Korobov), representing the commonly used remedies in Imperial Russia, as well as during the Soviet and post-Soviet periods. Finally, we traced the possible sources of veterinary knowledge related to those plants listed in Bloshenko et al. (59) which were reportedly used for ethnoveterinary purposes in the Soviet Union.

We found that the seeds of *Linum usitatissimum* were used for their mucolytic and diuretic properties, which is comparable with their recorded use for diarrhea, as flaxseed extract has been proven effective for treating enteric and non-enteric pathogens (60). These uses were earlier confirmed in Gurin (61), Rabinovich (62), and Korobov et al. (63), although the seeds were claimed to have a slight laxative effect. In addition, we recorded the use of *Linum usitatissimum* as a postpartum supplement, which has been found to be effective in rats (64) due to the presence of beneficial fatty acids (e.g., alpha-linolenic acid) as reported by Bloshenko et al. (59). Gurin (61) and Korobov et al. (63) also suggested the use of flax seeds in wet poultices to foster cicatrization. Flaxseeds were commonly sold in pharmacies during Soviet times. Flax used to be an important economic crop in Belarus, Pskov Oblast of Russia, and the Estonian part of Setomaa. During our fieldwork in Pskov Oblast, we often observed remnants of that time period on farms: retting ponds (*mochilo*) that were used in the cycle of fabric production from flax. Older informants could remember bringing flaxseed to the commonly accessible mills to produce flaxseed oil.

Bloshenko et al. (59) also reported the ethnoveterinary use of *Hypericum perforatum, Quercus robur*, and *Rumex* spp. for wound healing and cell regeneration, as well as for their antiseptic properties. *Hypericum perforatum* was also mentioned by Duke (65) who reported that in Soviet times it was used for several applications including the treatment of diarrhea, which we recorded in five of the countries we studied. Various uses of *Hypericum* were reported by Rabinovich (62) and Korobov et al. (63), but they were not mentioned by Gurin (61). However, this plant seems to have been known and used in folk medicine even before the Soviet Union [see (66)].

Deryabin and Tomalcheva (67) reported the use of *Quercus robur* for treating mild diarrhea (as we recorded in five countries), but they traced such a use back to the medieval European medicinal tradition. The use of *Quercus robur* was also recorded in Gurin (61), Rabinovich (62), and Korobov et al. (63). The roots of *Rumex* spp., and specifically *Rumex confertus*, were used in Russian traditional medicine as an astringent (68), use that we also recorded in the Russia and Bukovina. Its use has only been confirmed in Soviet and post-Soviet sources (62, 63).

We can therefore see some overlapping patterns in our records and the veterinary medicine promoted during the time of the Soviet Union, which established kolkhozes where livestock breeding was managed by highly specialized actors. Indeed, highly specialized veterinary doctors worked in almost every village. Several sources suggest that Soviet medicine relied largely on plants and products based on them and thus such uses were often transferred from the human sphere to the animal one (following the idea that livestock were considered to be part of the family). Although it was not explicitly stressed by our interviewees, we can see that some of our findings reflect the official medicinal and veterinary recommendations of the Soviet Union, possibly through the formation of veterinary specialists who actually treated the animals. The recommendations themselves relied on veterinary knowledge derived partially from ethnoveterinary knowledge that underwent scientific tests, so the local uses supported by the official veterinary medicine had a greater chance for survival.

Future research should investigate the heterogeneous trajectories that ethnoveterinary knowledge takes under different drivers of change to pastoral systems, and the complex networks of sources from where farmers derive their ethnoveterinary knowledge. Additionally, it would be interesting to evaluate the effects of the use of plant-based ethnoveterinary remedies in accordance with current legislation in Europe.

## Data Availability Statement

The original contributions presented in the study are included in the article/Supplementary Material, further inquiries can be directed to the corresponding author/s.

## Ethics Statement

The studies involving human participants were reviewed and approved by Ca' Foscari University of Venice Ethics Committee. Written informed consent for participation was not required for this study in accordance with the national legislation and the institutional requirements.

## Author Contributions

GM, GV, and RS conceived the study. NK, VK, OB, RS, RK, JP, NS, and GM gathered the data. RS, AP, and GV supervised the study. GM analyzed the data with the help of the co-authors. GM drafted the first version of the manuscript which was then reviewed, edited, and approved by OB, RK, VK, NK, JP, NS, AP, GV, and RS. All authors contributed to the article and approved the submitted version.

## Funding

This project has received funding from the European Research Council (ERC) under the European Union's Horizon 2020 research and innovation programme (grant agreement No. 714874).

## Conflict of Interest

The authors declare that the research was conducted in the absence of any commercial or financial relationships that could be construed as a potential conflict of interest.

## Publisher's Note

All claims expressed in this article are solely those of the authors and do not necessarily represent those of their affiliated organizations, or those of the publisher, the editors and the reviewers. Any product that may be evaluated in this article, or claim that may be made by its manufacturer, is not guaranteed or endorsed by the publisher.
