# Supplementary material for: Multifarious Trajectories in Plant-Based Ethnoveterinary Knowledge in Northern and Southern Eastern Europe
Source: Front Vet Sci. 2021 Oct 14;8:710019. doi: 10.3389/fvets.2021.710019 (PMC8551763; doi:10.3389/fvets.2021.710019)
Supplement: Supplementary file 1 [file Table_1.pdf]

Table 2 Plant taxa used in ethnoveterinary remedies for treating cattle. [W] Wild species; [C] Cultivated species; [B] Bought species; BL (Belarus); EE (Estonia); FI (Finland); LT (Lithuania); PL (Poland); RK (Russian Karelia); RO (Romania); RS (Russian Setomaa); UA (Ukraine). *No longer in use, number of use reports (UR) is indicated if more than one.

**Latin name and herbarium codes**	**Local Name**	**Part used**	**Preparation**	**Use**	**Use Report (*past)**
Achillea millefolium L. (Asteraceae) [W] NB 007; SB074; LE 01063441, LE 01063544	Coada șoricelului (RO); Деревій, тисячелетник (UA); Тысячелистник (RS)	Aerial parts (also Flowers only)	Infusion	Various problems	RO; 2 UA
				Stomach illnesses	RO
				Stomachache	RS*
				Diarrhea	3 UA
		Aerial parts	Tincture with alcohol	Diarrhea	UA
Aegopodium podagraria L. (Apiaceae) [W] LE 01063450, LE 01063430	Сныть (RS)	Aerial parts	Infusion	Urinary illnessesillnesses	RS*
Aesculus hippocastanum L. (Sapindaceae)	Каштан (UA)	Bark	Infusion	Diarrhea	2 UA
		Aerial parts	Infusion	Stomach illnessesillnesses	UA

[W]					
Allium cepa L.<br>(Amaryllidaceae)<br>[C]	Ceapă (RO); Sibul (EE); Лук (UA)	Bulbs	Soup with oil	Rumination problems	RO
			Fresh	Calving paralysis	EE*
				Mastitis	UA
Allium sativum L.<br>(Amaryllidaceae)<br>[C]	Usturoi (UA)	Bulbs	Fresh	Intestinal gas	2 UA
Alnus spp. including *A. glutinosa* (L.) Gaertn. and *A. incana* (L.) Moench<br>(Betulaceae) [W] LE 01063373; NB050	Вільха (UA)<br>Ольха (RK, RS); Alksnis, buziuliai (part used) (PL)	Cones; Flowers	Infusion; Decoction	Diarrhea	2 UA; 2 RK*<br>RK*<br>4 RS<br>PL*<br>PL*
Anethum graveolens L.<br>(Apiaceae)<br>[C]	Mărar (RO, UA), кріп (UA); Укроп (RK, RS)	Aerial parts	Infusion	Healthy	4 RO
				Postpartum supplement	RS*
				Calmant	2 UA
			Fresh	Postpartum supplement	RS

		Seeds	Dried	Stomach illnesses	UA
			Fresh	Gum illnesses	RK*
			Boiled	Postpartum supplement	RS*
Arctium spp. (Asteraceae) [W]	Лопух (BL)	Leaves	Topical application	Mastitis	BL*
Artemisia absinthium L. (Asteraceae) [W] SB005	Kietis paprastasis; Kartusis kietis, pialynas (LT); Pelin (RO); Pelynas (LT, PL); Палын, палынь (BL); Полынь (RK)	Aerial parts	Decoction; Infusion	Digestive illnesses	2 LT*
				Diarrhea	LT*; 3 PL; 3 BL*; RK
				Stomachache	LT*
				Strengthening	RO
				Stomach illnesses	RO; LT*; PL*
Artemisia vulgaris L. (Asteraceae) [W]	Pujo (FI)	Aerial parts	Boiled	Leg inflammation	FI*
Atropa belladonna L. (Solanaceae) [W]	Матриган (UA)	Roots	Fresh	Improving fertility	2 UA

Avena sativa L. (Poaceae) [C]	Овёс (RS)	Seeds	Fresh	Diarrhea	RS*
Beta vulgaris L. (Amaranthaceae) [C]	Raudonas burokėlis (LT); Sfeclă (RO)	Roots	Juice	Digestive problems	LT*
			Fresh	Healthy	2 RO
Betula spp. including *B. pendula* Roth (Betulaceae) [W] LE 01063357, LE 01063453; NB049; KAR12, KARDR10	Береза (UA); Берёза (RK, RS); Koivu (FI)	Buds	Dried	Diarrhea	UA
				Stomach illnesses	UA
		Wood (tar)	Topical application	Horn repair	RK*
				Wounds	RK*
			Fresh	Intestinal gas	RS*
		Twigs	Dried	Postpartum supplement	FI*
				Diuretic	FI*
Calendula officinalis L. (Asteraceae) [C]	Galbenele (RO); Ноготки, календула (RS); Medetkos (LT); Notkos (PL)	Aerial parts; Flowers	Ointment	Mastitis	RO
				Postpartum supplement	RO
			Tincture	Mastitis	RS *
			Decoction	Stomach illnesses	LT*
				Postpartum supplement	LT*

			Fresh	Miscarriage prevention	PL*
Calluna vulgaris (L.) Hull (Ericaceae) [W]<br><br>LE 01063447	Mox, kanarbik (RS)	Aerial parts	Infusion	Postpartum supplement	RS*
Cannabis sativa L.<br><br>(Cannabaceae) [C]	Каноплі (BL)	Seeds	Decoction	Supplement for calves	BL*
Carduus spp.<br><br>(Asteraceae) [W]	Čiartapalochas, dagys (LT)	Aerial parts	Buried under the threshold of the barn	Evil eye	LT*
				Anxiety	LT*
Carum carvi L.<br><br>(Apiaceae) [W]<br><br>SB007; NB037	Chimion (RO); Săcărică (RO, UA); Secărica (RO); Кмин, хміль (UA); Тмин (RS)	Aerial parts; Seeds	Infusion	Diarrhea	8 RO; 3 UA
				Healthy	RO
				Postpartum supplement	UA; RO; RS*
				Stomach illnesses	RO; 3 UA
				Abdominal pain	UA
			Fresh	Improving fur	2 UA
		Latex	Locally applied	Blisters on utters	RO

Chelidonium majus L. (Papaveraceae) [W]<br><br>SB003	Rostopască (RO); Ugniažolė (LT); Чыстацел (BL)	Aerial parts	Decoction mixed with beetroot juice	Indigestion	LT*
		Leaves	Decoction	Diarrhea	BL*
Coffea arabica L. and C. *canephora* Pierre ex A.Froehner.<br><br>(Rubiaceae) [B]	Kava (PL)	Seeds	Infusion	Diarrhea	PL*
Coriandrum sativum L.<br><br>(Apiaceae) [C]	Coriandru (RO)	Seeds	Infusion	Diarrhea	2 RO
Epilobium angustifolium L. (Onagraceae) [W]	Иван-чай (RS)	Aerial parts	Concoction	Diarrhea	RS*
Equisetum arvense L.<br><br>(Equisetaceae) [W]<br><br>SB020	Coada calului, padivolos (RO); Barbaursuli (UA)	Aerial parts	Infusion	Diarrhea	2 RO
				Appetite stimulant	RO
				Stomach illnesses	2 UA
				Improving fertility	RO
Fraxinus excelsior L.<br><br>(Oleaceae) [W]	Ясен (UA)	Bark	Boiled	Diarrhea	UA

Gymnocarpium dryopteris (L.) Newman (Cystopteridaceae) [W]	Uročnikas (LT)	Aerial parts	Smoked	Evil eye	LT*
Helianthus annuus L.<br><br>(Asteraceae) [C]	Päevalill (EE); Соняшник (UA)	Seeds	Oil	Intestinal gas	2 EE*
				Diarrhea	3 UA
Hordeum vulgare L.<br><br>(Poaceae) [C]	Ячмень (RS); Orz (RO)	Seeds	Fresh	Diarrhea	RS*
			Boiled	Diarrhea	RO
Hypericum spp. including H. *perforatum* L. (Asteraceae) [W]<br><br>SB068; NB005;<br><br>LE 01063483,<br><br>LE 01063443, LE 01063428	Зверобой (RK; RS); Звіробій (UA), зверобой (UA, BL); Pojarniță, Sunătoare (RO)	Aerial parts; Whole plant	Infusion; Decoction	Diarrhea	RK*; 8 UA; 6 RO; RS*; BL
				Various diseases	3 UA; RO*
				Stomach illnesses	6 UA; 4 RO
				Intestinal gas	2 UA; RO
				Inappetence	2 UA; RS*
				Abdominal pain	UA

				Postpartum supplement	UA; 2 RO
				Panacea	RO
				Anti-inflammatory for the intestines	RS*
Inula helenium L. (Asteraceae) [C]	Deviasylas (LT)	Roots	Infusion or decoction	Stomach illnesses	LT*
Ledum palustre L. (Ericaceae) [W]	Gailis, bahaunyk (LT)	Aerial parts	Dried or fresh	Pest prevention	LT*
Levisticum officinale W.D.J.Koch (Apiaceae) [C]	Starėdup (LT); Любисток (UA)	Roots	Infusion or decoction	Stomach illnesses	LT*
		Leaves	Infusion	Intestinal gas	UA
Linum usitatissimum L. (Linaceae) [C]	Лён (BL; RS); Linas, linų sėmenys [part used] (LT); In (RO); Лен, лень, льон (UA); Pellava (FI); Lina (EE)	Seeds	Decoction; Infusion; Concoction	Diarrhea	2 BL; 4 RS*
				Various illnesses	LT*
				Stomach illnesses	LT*
				Postpartum supplement	6 EE*; 2 RS; 4 UA
				Intestinal gas	2 EE*; RS*

				Health strengthening	EE*
				Rumination illnesses	RO
			Cooked in milk	Afterbirth supplement	LT*
			Dried	Fever	UA
			Fresh	Postpartum supplement	FI*
				Health strengthening	EE*
Malva verticillata L. (Malvaceae) [C]	Кудрявець (UA)	Aerial parts	Infusion	Postpartum supplement	2 UA
Matricaria chamomilla L. (Asteraceae) [W/C] SB019	Mușețel (RO); Romaniță (RO, UA); Ромашка (UA, RS)	Aerial parts Whole plant	Infusion	Stomach illnesses	4 UA
				Abdominal pain	UA
				Cleansing	2 UA
				Diarrhea	UA; RO
				Eye washing	2 RO
				Panacea	RO
				Postpartum supplement	2 RO; UA
			Infusion	Various illnesses	EE*

Matricaria discoidea DC. (Asteraceae) [W]<br><br>LE 01063395, LE 01063444, LE 01063416; S005; SE017, SE66	Morokummel, kodukummel (EE); Kummel, ромашка (RS)	Aerial parts	Boiled	Diarrhea	RS*
			Concoction	Urinary inflammation	RS*
		Flowers	Infusion	Disinfecting	RS*
Mentha spp.<br><br>(Lamiaceae) [W/C]<br><br>SB096	Mentă (RO); Šalta mėta (LT)	Aerial parts	Infusion	Postpartum supplement	2 RO
				Stomach illnesses	LT*
Nicotiana tabacco L. (Solanaceae) [C]	Тютюн (UA)	Aerial parts	Boiled	Mastitis	UA
Picea spp. (incl. *P. abies* (L.) H.Karst. [W] (Pinaceae)	Ель (RK)	Twigs	Infusion	Diarrhea	RK*
Pinus sylvestris L. (Pinaceae) [W]<br><br>LE 01063372;<br><br>SE120	Mänd (EE); Сосна (RK)	Twigs	Fresh	Vitamin supplement	EE*
			Infusion	Diarrhea	RK*
Plantago lanceolata L.	Подорожник (UA)	Roots	Infusion	Diarrhea	UA

(Plantaginaceae) [W]					
Plantago major L.<br>(Plantaginaceae) [W] NB022	Подорожник (UA)	Aerial parts	Infusion	Stomach illnesses	UA
Potentilla erecta (L.) Raeusch.<br>(Rosaceae) [W]<br>LE 01063358	Калган (RK)	Roots	Dried, decoction	Diarrhea	2 RK*
Quercus robur L. (Fagaceae) [W]<br>LE 01063338,<br>LE 01063451; SE100	Tamm (EE); Дуб (RS, RK, UA); Ąžuolas (PL)	Bark	Boiled; Concoction; Decoction; Infusion	Diarrhea	EE; 4 RS; 3 UA; 3 PL; 2 RK*
				Stomachache	RS*
				Stomach illnesses	PL*
Rumex spp. (acidic) (incl. *R. acetosa* L. LE 01063414 and *R. acetosella* L.)<br>(Polygonaceae) [W]	Кислица (RS); щавель (RS, UA), щіва (UA)	Aerial parts; Roots	Infusion	Diarrhea	RS*; 4 UA
Rumex spp. (non-acidic) including	Stevie (RO); Steja, Steje (RO,	Aerial parts;	Infusion	Diarrhea	5 UA; 8 RO; 2

R. alpinus L. and *R. confertus* Willd.<br><br>(Polygonaceae) [W]<br><br>SB067; NB003; NB081;<br><br>LE 01063514 (*Rumex crispus* L.)<br><br>LE 01079357 (*Rumex thyrsiflorus* Fingerh.)	UA); Шіва (UA); Конский щавель (RK, RS)	Seeds; Whole plant; Roots			RK*; 4 RS*
		Aerial parts	Infusion	Postpartum supplement	2 RO
				Stomach illnesses	3 UA
Secale cereale L.<br><br>(Poaceae) [C]	Рожь (RS)	Seeds	Fresh	Diarrhea	RS*
Sorbus aucuparia L.<br><br>(Rosaceae) [W]<br><br>LE 01063446	Рябина (RS)	Fruits	Fermented (wine)	Diarrhea	RS*
Syringa vulgaris L.<br><br>(Oleaceae) [C]<br><br>LE 01063458	Сирень (RS)	Leaves; Flowers	Infusion	Diarrhea	2 RS
Tanacetum vulgare L.	Пижма (RK)	Aerial parts	Decoction	Intestinal gas	RK*

(Asteraceae) [W]<br><br>LE 01063374					
Tilia cordata Mill.<br><br>(Malvaceae) [W]<br><br>SB017	Liepa (PL); Tei (RO)	Bark (inner layer)	Kneaded with bread and fed to cows	Rumination problems	PL*
		Flowers	Infusion	Improving digestion	2 RO
				Stomach illnesses	2 RO
Triticum aestivum L.<br><br>(Poaceae) [C]	Пшеница (RS)	Seeds	Fresh	Diarrhea	RS*
Urtica dioica L. (Urticaceae) [W]<br><br>LE 01063363,<br><br>LE 01063436; KAR02	Nokkonen, čiiloiheinä, vihulainen (FI); Крапива (RS, RK)	Aerial parts	Boiled	Leg inflammation	FI*
				Panacea	FI*
			Infusion	Diarrhea	RK*, RS*
			Topical application	Mastitis	RS*
Vaccinium myrtillus L.<br><br>(Ericaceae) [W]<br><br>LE 01063348; NB060	Черника, must'oj (RK); Афинник (UA)	Fruits	Dried	Diarrhea	RK*
				Stomach illnesses	UA

Vaccinium vitis-idaea L. (Ericaceae) [W]<br><br>NB061	Merişor (UA)	Fruits	Infusion	Stomach illnesses	UA

Table 3 Plant taxa used in ethnoveterinary remedies for treating other livestock (besides cattle). [W] Wild species; [C] Cultivated species; BL (Belarus); EE (Estonia); FI (Finland); LT (Lithuania); PL (Poland); RK (Russian Karelia); RO (Romania); RS (Russian Setomaa); UA (Ukraine). *No longer in use, number of use reports (UR) is indicated if more than one.

**Latin name and family**	**Local Name**	**Part used**	**Preparation**	**Use**	**Treated animal**	**Use reports per country**<br><br>**(*past)**
Achillea millefolium L. (Asteraceae) [W]<br><br>LE 01063356;<br><br>NB007	Kraujažolės (PL); Тысячелистник (RK); Деревій (UA)	Aerial parts	Fresh	Healthy	Turkeys	LT*
			Decoction; Infusion	Stomach illnesses	Various animals including piglets	2 UA; RK*
				Diarrhea	Various animals	2 UA

Allium cepa L. (Amaryllidaceae) [C]	Лук (RS)	Bulbs	Fresh	Postpartum supplement	Various animals	RS*
Alnus spp. including *A. incana* (L.) Moench<br><br>(Betulaceae) [W]<br><br>LE 01063373	Ольха (RS, RK); Alksnis, buziuliai (part used) (PL)	Cones	Decoction; Infusion; Concoction	Diarrhea	Piglets, various animals	2 RK*; PL*; 3 RS*
Artemisia absinthium L. (Asteraceae) [W]	Palynas, Pialynas (BL); Pelynas (LT, PL, BL); Kietis (LT); Pelynai (PL)	Aerial parts	Decoction	Diarrhea	Various animals including pigs	2 BL; 2 LT*; PL*
				Digestive illnesses	Pigs	LT
				Inappetence	Pigs, various animals	LT; BL*
			Mixed into sugary syrup; Smoked; Fresh	Pest prevention	Bees, various animals	PL; 3BL, 2 LT
Avena sativa L. (Poaceae) [C]	Овёс (RS)	Seeds	Fresh	Diarrhea	Pigs	RS*

Betula spp. (Betulaceae) [W] LE 01063357, LE 01063453; KAR12	Берёза (RK), koivu (FI; RK)	Wood (tar)	Topical application	Horn/Hoof repair	Various animals	RK*
		Bark	Topical application	As a plaster for broken legs	Sheep	FI*
		Twigs	Fresh	Promoting tooth replacement	Various animals	FI*
			Dried	Stomach illnesses	Sheep	FI*
Calendula officinalis L. (Asteraceae) [C]	Нагаткі (BL); Medetkos (LT)	Flowers	Smoked	Evil eye	Various animals	BL*
			Decoction	Various illnesses	Various animals	LT*
Carum carvi L. (Apiaceae) [W] NB037	Sacarica (UA)	Aerial parts	Infusion	Inappetence	Chickens	2 UA
Chelidonium majus L. (Papaveraceae) [W]	Čistatiela (BL)	Aerial parts or whole plant	Smoked	Pest prevention	Bees	BL
			Decoction	Animal cleansing	Various animals	2 BL*

Chenopodium album L. (Amaranthaceae) [W]	Balanda (PL; LT)	Aerial part	Fresh	Healthy	Geese, Pigs	PL*; LT*
Cyanus segetum Hill. (Asteraceae) [W]	Vosilkos (LT)	Flowers	Decoction	Various illnesses	Various animals	LT*
Daphne mezereum L. (Thymelaceae) [W]	Näsiä (FI)	Fruits	Dried, used in a cream mixed with other ingredients	Abscess; Inflammation	Various animals	2 FI
Equisetum arvense L. (Equisetaceae) [W]	Пупыши (RS)	Strobilus	Fresh	Vitamin supplement	Pigs, Sheep	2 RS*
Fagus sylvatica L. (Fagaceae) [W] SB060	Fag (RO)	Bark	Decoction	Healthy	Various animals	2 RO
Filipendula ulmaria (L.) Maxim. (Rosaceae) [W] SE041	Angervaks (EE)	Flowers	Infusion	Supplement	Various animals (mostly cows)	EE*
Frangula alnus Mill. (Rhamnaceae) [W]	Hundimarjad, soepuu marjad, paakspuu (EE)	Fruits	Juice	Hive disinfection	Bees	EE
Hordeum vulgare L.	Ячмень (RS)	Seeds	Fresh	Diarrhea	Pigs	RS*

(Poaceae) [C]						
Hypericum spp. (Hypericaceae) [W]<br><br>SB068<br><br>LE 01063443, LE 01063428; SE059, SE002, SE003	Sacarica (RO); Naistepuna (EE); Зверобой (RS)	Aerial parts	Infusion; Concoction	Inappetence	Chickens	2 RO
				Supplement	Various animals (mostly cows)	EE*
				Intestinal anti-inflammatory	Pigs	RS*
				Healthy	Various animals	RS*
Juniperus communis L.<br><br>(Cupressaceae) [W]<br><br>SE116	Jėglys (BL); Ėglis, kadagys (LT); Mošovelnik, kadakas (EE)	Twigs	Smoked	Evil eye	Various animals	BL*; LT*
			Decoction	Mineral supplement	Various animals	EE*
Lamium album L.<br><br>(Urticaceae) [W]	Bielaja krapiva (BL)	Aerial part	Decoction	Inappetence	Various animals	BL*
Ledum palustre L. (Ericaceae) [W]<br><br>SE038	Gailis (PL); Baūnas, Gailius (LT); Sookail (EE)	Aerial part, leaves	Fresh	Digestive illnesses	Pigs	PL*
				Pest repellent	Various animals	LT*

				Erysipelas	Pigs	EE*
Linum usitatissimum L. (Linaceae) [C]	Лён (RS)	Seeds	Concoction	Diarrhea	Pigs	RS*
Nicotiana rustica L.<br><br>(Solanaceae) [C]	Tabakas (LT); Tubakas (EE)	Aerial parts; Leaves	Fresh	Pest prevention	Bees	LT
			Decoction topically applied	Viper bites	Various animals (mostly cows)	EE*
Origanum vulgare L. (Lamiaceae) [W]<br><br>SB036	Şovârf (RO)	Whole plant	Infusion	Stomach illnesses	Various animals	RO
Papaver somniferum L.<br><br>(Papaveraceae) [C]	Aguonos (PL)	Leaves	Fresh	Calmant	Geese	PL*
Picea abies (L.) H.Karst. (Pinaceae) [W]<br><br>KAR06a	Kuusi (FI)	Resin	Fresh	Wounds	Various animals	2 FI*
Pinus spp. including *P. sylvestris* (Pinaceae) [W]	Mänty, petäjä (FI); Mänd (EE);	Resin	Burnt	Sneezing	Horses	FI*
			Fresh	Wounds	Various animals	FI*; 2 EE*

LE 01063372; KARDR24, SE120	Сосна (RK); Хвоя (BL)	Wood (tar); Roots	Burnt	Hoof healing	Horses	FI*
				Wounds	Various animals including horses	3 FI*
		Thorns	Infusion	Cough	Horses	EE
			Decoction	Vitamin supplemen t	Dogs	RK*
		Twigs	Decoction	Suppleme nt	Bees	BL
Potentilla erecta L. (Rosaceae) [W] LE 01063358	Калган (RK)	Roots	Infusion, Decoction	Diarrhea	Pigs, various animals	3 RK*
Prunus padus L. (Rosaceae) [W] LE 01063361	Черёмуха (RS, RK)	Fruits	Infusion	Diarrhea	Sheep	RK*
				Stomach illnesses	Sheep	RS*
Pteridium aquilinum (L.) Kuhn (Dennstaedtiaceae) [W]	Paparčiai (LT)	Aerial parts	Dried, laid in barns	Pest repellent	Various animals	LT*

Quercus robur L. (Fagaceae) [W] LE 01063338 LE 01063451; SE100	Aržuolas, Ąžuolas (PL); Дуб (RS, RK); Tamm (RS; EE)	Bark	Infusion; Decoction	Diarrhea	Various animals, pigs	PL*, 4 RS, EE, RK*
		Twigs	Smoked	Evil eye	Various animals	LT*
Rumex spp. including *R. confertus* Willd. (Polygonaceae) [W] SB067; LE 01063383, LE 01063513, LE 01063540, LE 01063479	Конский щавель (RK; RS); Щавель (RK); Ștevie (RO)	Aerial parts; Leaves; Root	Infusion	Diarrhea	Pigs, various animals	3 RK*; 2 RS; 2 RO
Ruta graveolens L. (Rutaceae) [W]	Rūta (PL)	Aerial parts	Decoction	Fleas	Cats, Dogs	PL*
Salix spp. (Salicaceae) [W]	Vierba (BL); Verba, Karklas (LT)	Twigs	Consecrated	Evil eye	Various animals	BL*; LT*
Secale cereale L. (Poaceae) [W]	Рожь (RS)	Seeds	Fresh	Diarrhea	Pigs	RS*
Silene viscaria (L.) Jess.	Tõrvalill (EE)	Aerial parts	Infusion	Suppleme nt	Various animals	EE*

(Caryophillaceae) [W]					(mostly cows)	
Solanum tuberosum L. (Solanaceae) [C]	Картофель (RK)	Tubers	Starch	Diarrhea	Pigs	RK*
Stellaria media (L.) Vill. (Caryophyllaceae) [W]	Žliūgė (PL; LT)	Aerial parts	Fresh	Healthy	Geese, Pigs	PL*; LT*
Symphytum officinale L. (Boraginaceae) [W]	Živakastas (BL)	Roots	Decoction	Dislocatio ns	Various animals	BL*
Syringa vulgaris L. (Oleaceae) [C] LE 01063458	Сирень (RS)	Flowers	Infusion	Diarrhea	Pigs	RS*
Tanacetum vulgare L. (Asteraceae) [W] LE 01063374	Пижма (RK); Pižma (BL)	Aerial part	Infusion	Diarrhea	Pigs	RK*
			Smoked	Pest prevention	Bees	BL
Taraxacum officinale (L.) Webb ex F.H.Wigg. (Asteraceae) [W] LE 01063354	Одуванчик (RK)	Roots	Dried	Various illnesses	Sheep	RK*

Trifolium repens L.<br><br>(Leguminosae) [W]	Dobilas (LT)	Flowers	Decoction	Various illnesses	Various animals	LT*
Triticum aestivum L.<br><br>(Poaceae) [C]	Пшеница (RS)	Seeds	Fresh	Diarrhea	Pigs	RS*
Urtica dioica L. (Urticaceae) [W]<br><br>LE 01063436	Крапива (RS); Dilgėlės (LT); Dilgėlė (BL; LT; PL); Dzirgėlė (PL)	Aerial parts; Whole plant	Concoction; Dried; Fresh	Healthy	Various animals, pigs, turkeys	RS*: 3 LT*; BL*: PL*
				Vitamin supplemen t	Chickens	RS
		Roots	Decoction	Preventing intestinal parasites	Pigs	LT*
Urtica urens L. (Urticaceae) [W]	Dilgėlė (LT)	Shoots	Fresh	Healthy	Pigs	LT*
